# Modeling transport of antibiotic resistant bacteria in aquatic environment using stochastic differential equations

**DOI:** 10.1038/s41598-020-72106-3

**Published:** 2020-09-15

**Authors:** Ritu Gothwal, Shashidhar Thatikonda

**Affiliations:** Department of Civil Engineering, Indian Institute of Technology Hyderabad, Kandi, Sangareddy, Telangana 502285 India

**Keywords:** Environmental sciences, Environmental chemistry, Environmental impact

## Abstract

Contaminated sites are recognized as the “hotspot” for the development and spread of antibiotic resistance in environmental bacteria. It is very challenging to understand mechanism of development of antibiotic resistance in polluted environment in the presence of different anthropogenic pollutants. Uncertainties in the environmental processes adds complexity to the development of resistance. This study attempts to develop mathematical model by using stochastic partial differential equations for the transport of fluoroquinolone and its resistant bacteria in riverine environment. Poisson’s process is assumed for the diffusion approximation in the stochastic partial differential equations (SPDE). Sensitive analysis is performed to evaluate the parameters and variables for their influence over the model outcome. Based on their sensitivity, the model parameters and variables are chosen and classified into environmental, demographic, and anthropogenic categories to investigate the sources of stochasticity. Stochastic partial differential equations are formulated for the state variables in the model. This SPDE model is then applied to the 100 km stretch of river Musi (South India) and simulations are carried out to assess the impact of stochasticity in model variables on the resistant bacteria population in sediments. By employing the stochasticity in model variables and parameters we came to know that environmental and anthropogenic variations are not able to affect the resistance dynamics at all. Demographic variations are able to affect the distribution of resistant bacteria population uniformly with standard deviation between 0.087 and 0.084, however, is not significant to have any biological relevance to it. The outcome of the present study is helpful in simplifying the model for practical applications. This study is an ongoing effort to improve the model for the transport of antibiotics and transport of antibiotic resistant bacteria in polluted river. There is a wide gap between the knowledge of stochastic resistant bacterial growth dynamics and the knowledge of transport of antibiotic resistance in polluted aquatic environment, this study is one step towards filling up that gap.

## Introduction

Rampant use of antibiotics caused antibiotic pollution in natural aquatic environment such as rivers, lakes, groundwater, seawater, sediments, plants and aquatic animals^12,21,26,28,29,39,52^. Continuous presence of traces of antibiotics in environment lead to the rapid development and spread of antibiotic resistance, consequently threatening the effectiveness of antibiotics. Antibiotics enter aquatic environment majorly through effluent discharges from wastewater treatment plants^1,26,31,36,56^. After entering the natural environment antibiotics get subjected to various ecological/environmental processes such as advection, dispersion, diffusion, degradation, settling, resuspension, pH, sorption, sunlight, temperature, presence of organic compounds/minerals, and population of bacteria. Ecological factors affect the fate of antibiotics significantly, hence play important role in the occurrence of antibiotic resistance in environment. For example, sorption dictates the uptake and degradation of antibiotics over the reaches of streams as antibiotics are very much susceptible to getting adsorbed to bed material. Sorption behavior of antibiotics is dependent on their molecular structure and physicochemical properties of sediments and water, such as pH, organic matter and mineral contents^3,9,10,42,48,50^. Sunlight is another example of ecological factor which affects the presence of antibiotics through photodegradation, which is again dependent on other environmental factors such as pH, temperature, presence of salts, organic compounds etc.^23,26,51^.

Mathematical models play a key role in understanding and predicting natural phenomena along with their uncertainties^44^. There are mathematical models for predicting antibiotic concentrations in aquatic environment both spatially and temporally^13,18,38^. Predicting antibiotic resistance in the hospital environment has been studied by^2,15,32,55^. However, very few studies have attempted to model the transport of antibiotics and its resistance in aquatic environment. There is a limited knowledge of the prediction of antibiotic resistance associated with microbes in an aquatic environment, as it is a complex ecosystem considering the wide range of selection pressures and transmission pathways. Hellweger et al.^19^ developed a simple model of tetracycline resistance in application to Poudre River (Colorado) to find the cause of resistance while considering the effect of tetracycline on growth rate of bacteria. We have also developed a conceptual and mathematical model for steady state one dimensional advection–dispersion dominated transport of fluoroquinolone and its resistant culture in application to the Musi river, South India^13^. These models comprise several partial differential equations representing physical processes such as advection, dispersion, adsorption, degradation, settling, re-suspension, diffusion, bacterial growth, plasmid conjugation and segregation. The models include state variables such as fluoroquinolone, total suspended particles, organic matter, heavy metals, antibiotic resistant bacteria, and susceptible bacteria.

In a transport model the variables are affected by several natural phenomenon. For example, total suspended particles are affected by hydrological conditions such as river bed slope, type of bed material, flow of water, depth of water and rainfall, and also, due to ecosystem of the river as well as anthropogenic factors^8,10,33^. Similarly, other variables are also dependent on the number of natural processes. All the variables which affect a model should be considered for accurate prediction of model results. However, the variables in the model are also the sources of randomness due to which uncertainty creeps in the model. Uncertainty in a model may be attributed to randomness in model parameters, initial boundary and end boundary conditions. It is important not to ignore uncertainty in the model in order to obtain predictable outcomes of the studied system. In this study, our goal is to analyze the impact of stochasticity in the model parameters on temporal and spatial prediction of antibiotic resistance in aquatic environment.

## Methods

### Deterministic model review

**Figure 1:**
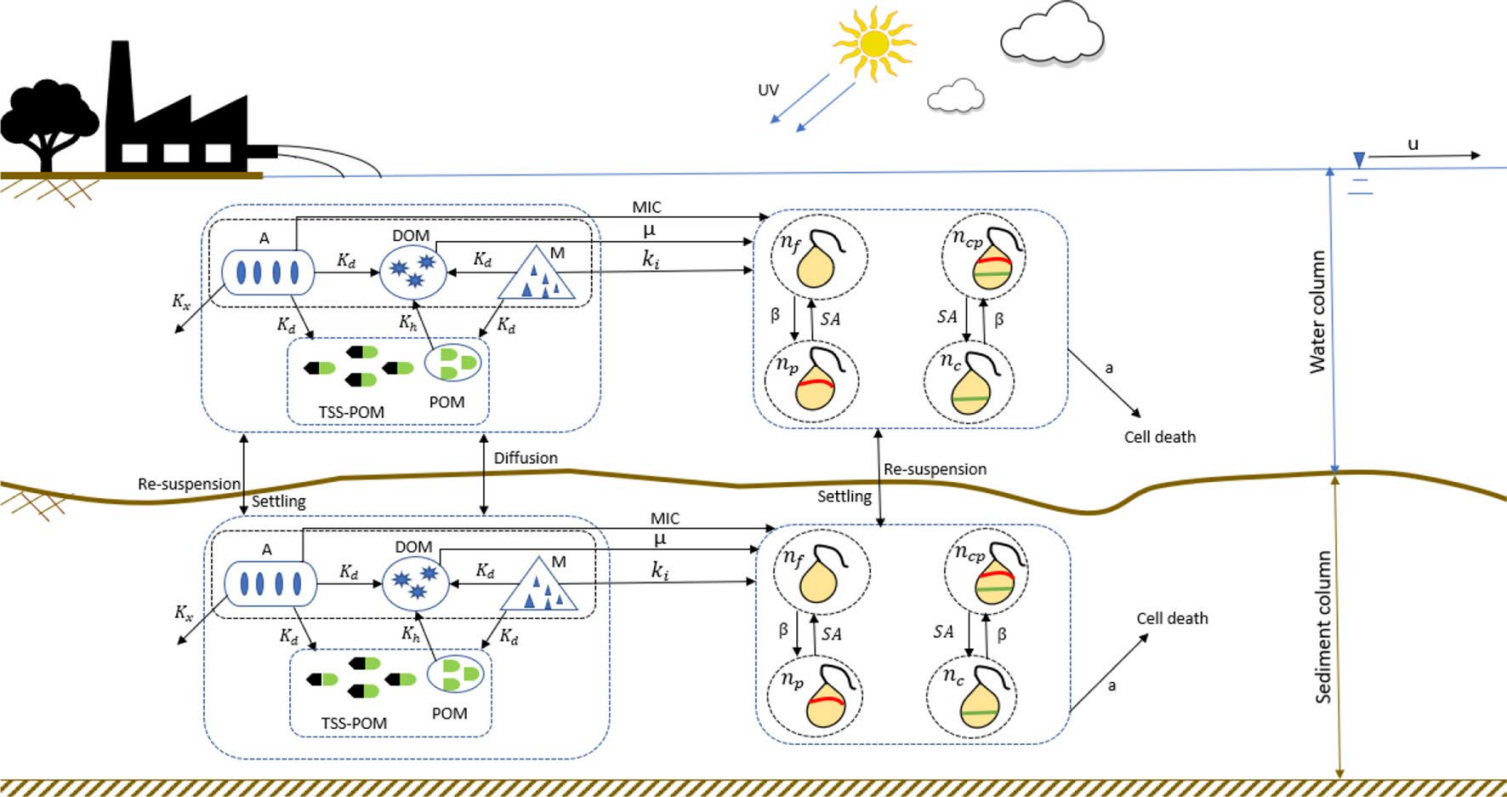
Schematic diagram of model state variables and processes in the water column and sediment column.

The deterministic model represents one-dimensional advection dispersion transport of fluoroquinolones and its resistant bacteria in the aquatic environment of Musi river (India). Schematic diagram of the model is shown in Fig. 1. Concentrations of fluoroquinolone (A), heavy metal (M), total suspended solid (TSS), particulate organic matter (POM), dissolved organic matter (DOM) and population of bacteria (N), are the state variables used in this model. The bacterial culture population is categorized as: free culture with no resistance (${\mathrm{n}}_{f}$), resistant culture with gene carried on the plasmid (${\mathrm{n}}_{p}$); resistant culture with resistant gene carried on chromosome (${\mathrm{n}}_{c}$); and the culture having both resistances on plasmid as well as chromosome (${\mathrm{n}}_{cp}$); the total population of bacteria culture is given by $\text{N} = {\mathrm{n}}_{f}{+\mathrm{n}}_{p}{+\mathrm{n}}_{c}+{\mathrm{n}}_{cp}$.

The partial differential equations are developed from mass balance around a control volume of A Δx. All the contaminant concentrations in sediment and water are referred to the mass per unit of total environmental volume (liters). The mass balance equations are comprised of the transport terms and reaction terms. The transport terms are advection, dispersion, settling, re-suspension and diffusion. Reaction terms in the model are adsorption, hydrolysis, bacterial growth, bacterial growth inhibition, death of bacteria, degradation, resistance gene transfer (conjugation) and loss of resistance gene (segregation).

$$ {\text{Accumulation}} = \underbrace {{{\text{Inputs}} - {\text{Outflows}}}}_{{{\text{Transport}}}} \pm {\text{Reactions}} $$

The chemical, physical, and ecological reactions are affected due to reaction rate constants. The reaction rate constants used in the model are enlisted in Table 1.

**Table 1:** Summary of parameters included in the model.

S. no.	Constant	Definition	Unit	Value	Minimum	Maximum	References
1	${\mathrm{K}}_{set}$	Constant for settling (settling velocity/mean depth, Vs/h)	/h	0.02	0.002	0.135	^33^
2	${\mathrm{K}}_{dsolidA}$	Solids partition coefficient with antibiotics	l/mg	0.0002	0.00007	0.005	^10^
3	${\mathrm{K}}_{dDOMA}$	DOM partition constant with antibiotics	l/mg	0.002	0.00007	0.005	^10^
4	${\mathrm{K}}_{dsolidM}$	Solids partition coefficient with metals	l/mg	0.000002	0.000002	0.0002	^8^
5	${\mathrm{K}}_{dDOMM}$	DOM partition constant with metals	l/mg	0.000002	0.000002	0.0002	^8^
6	${\mathrm{K}}_{resus}$	Constant for resuspension (resuspension velocity/mean depth of water layer attached to bottom, Vr/ho)	l/mg	0.00000239			^19^
7	${\mathrm{K}}_{diff}$	Diffusion constant (resuspension velocity/mean depth of water layer attached to bottom, Vd/ho) (ho = 0.1 m = 10 cm)	m/h	0.000208	0.00004167	0.0004167	^49^
8	${\mathrm{K}}_{xawc}$	Constant for degradation of antibiotics in water column	/h	0.05	0.001917	1.7916	^12^
9	${\mathrm{K}}_{xased}$	Constant for degradation of antibiotics in sediments	/h	0.03	0.000958	0.01	^12^
10	${\mathrm{K}}_{Hwc}$	Hydrolysis rate constant for particulate organic matter in water column	/h	0.00208	0.000416	0.0029	^19^
11	${\mathrm{K}}_{Hsed}$	Hydrolysis rate constant for particulate organic matter in sediment	/h	0.0002	0.00416	0.0029	^19^
12	Porosity	Porosity		0.3			^20^
13	a	Extrinsic density-dependent death rate of cells	/h	0.0006	0.0001	0.00625	^7^
14	SA	Rate of segregation	/h	0.000001	0.00000104	0.0054	^17^
15	beta	Rate of horizontal transfer of plasmid	/h	0.000045	0	1.0	^14^
16	${\mathrm{Y}}_{f}$	yield coefficient of wild-type cells	mg/mg	0.4	0.2	0.52	^7^
17	${\mathrm{Y}}_{p}$	yield coefficient of bacterial cells with resistance carrying the plasmid	mg/mg	0.3	0.2	0.52	^7^
18	${\mathrm{Y}}_{c}$	yield coefficient of bacterial cells with resistance on chromosomes	mg/mg	0.3	0.2	0.52	^7^
19	${\mathrm{Y}}_{cp}$	yield coefficient of bacterial cells with resistance on both plasmid as well as chromosome	mg/mg	0.2	0.2	0.52	^7^
20	η	mg of metal reduced per gm of substrate utilized by bacteria	mg/mg	0.01			^43^
21	${\upmu }_{maxwc}$	the maximum specific growth rate in the water column	/h	0.1083	0.01083	0.1875	^7^
22	${\upmu }_{maxsed}$	the maximum specific growth rate in sediment	/h	0.0108	0.009	0.1875	^7^
23	${\mathrm{K}}_{s}$	the half rate constant	mg/l	9.1	0.2	18	^7^
24	${\mathrm{K}}_{im}$	metal inhibition rate constant	mg/l	3.049			^43^
25	${\mathrm{C}}_{c}$	Cost of resistant gene when carried on chromosome		0.02	0	1.2	^5^
26	${\mathrm{C}}_{p}$	Cost of resistant gene when carried on plasmid		0.05	0	1.2	^5^
27	X	cost plasmid carriage		0.01	0	1.2	^5^
28	MIC	Minimum inhibitory concentration	mg/l	4	0.05	8	^40^

Mass balance equations are presented without transport terms and single term is used to represent resistant bacterial culture as ${n}_{resistant}$, to simplify the model for clarity. Full equations are mentioned in supplementary information.

- Fluoroquinolones
  Fluoroquinolones are majorly degraded due to photolysis and a little due to oxidation, hence first ordered decay was assumed for antibiotics in this model.
  $$\frac{\partial A}{\partial t}={- K}_{xa }A$$
- Metals
  Metals utilization is considered for the growth of bacteria up to a certain concentration and is treated as inhibitor above that inhibition level of concentration. No other decay was assumed for the metals.
  $$\frac{\partial M}{\partial t}=- \eta \left(\frac{{\mu }_{f }{n}_{f}}{{Y}_{f}}+\frac{{\mu }_{resistant }{n}_{resistant}}{{Y}_{resistant}}\right)$$
- Total suspended solids
  Total suspended solids comprise of organic and inorganic fractions and only organic fraction is subjected to the hydrolysis.
  $$\frac{\partial TSS}{\partial t}=-{ZK}_{H }TSS$$
- Particulate organic matter
  Particulate organic matter is the fraction of total suspended solids and acts as a source for dissolved organic matter due to hydrolysis.
  $$\frac{\partial POM}{\partial t}=\left(a{n}_{f}\right)+\left(a{n}_{resistant}\right)$$
- Dissolved organic matter
  Dissolved organic matter acts as a substrate for the bacteria. In this model the total DOM is assumed to be available for the bacteria.
  $$\frac{\partial DOM}{\partial t}=-\left(\frac{{\mu }_{f }{n}_{f}}{{Y}_{f}}+\frac{{\mu }_{resistant }{n}_{resistant}}{{Y}_{resistant}}\right)$$
- Susceptible and resistant culture
  Apart from transport terms the concentrations of bacterial culture changes with the function of growth and respiration. The concentration of bacteria is defined on a biomass basis (mg/l) which is a usual practice in biogeochemical transport models^7,19^. The death rate of bacteria is represented by ‘a’.
  $$ \frac{{\partial n_{f} }}{\partial t} = \left( {\left( {1 - \frac{N}{{N_{max} }}} \right)n_{f} .\mu_{f} } \right) - \left( {a.n_{f} } \right) - \left( {\beta \frac{{n_{f} n_{resistant} }}{{n_{f} + n_{resistant} }}} \right) + SA.n_{resistant} $$
  The growth of resistant culture is affected due to exchange of mobile resistant gene among bacteria population. The rate of resistant gene transfer on plasmid is represented with β and rate of losing resistant gene on plasmid is also known as “segregation”, represented with SA^25,46^.
  $$ \frac{{\partial n_{resistant} }}{\partial t} = \left( {\left( {1 - \frac{N}{{N_{max} }}} \right)n_{resistant} .\mu_{resistant} } \right) - \left( {a.n_{resistant} } \right) + \left( {\beta \frac{{n_{f} n_{resistant} }}{{n_{f} + n_{resistant} }}} \right) - SA.n_{resistant} $$

### Stochastic model formulation

Stochastic process or random process is a collection of random variables representing the evolution of random values over time in some system. In a deterministic process the same trajectory of outcome is observed for a given set of initial conditions and parameter values, but in a stochastic process there is some indeterminacy, and the process may evolve in several directions. Hence, stochastic partial differential equation arises when randomness is introduced into the phenomena represented by a deterministic differential partial equation in a meaningful way, and relevant parameters are modeled as a suitable stochastic process^35^.

The SPDE model is comprised of two parts: first is deterministic part, and the other is probabilistic part whose dynamics are partly driven by noise terms. Deterministic part of the variable and its stochastic fluctuation controlled the process rate at time step Δt. SPDEs are solved similarly as PDE, but the noise term $\xi_{i}$ is calculated by randomly drawing a number from the standard normal distribution between 0 to 1 at each Δt. Each random draw is scaled by Δt as $\xi_{i} = \frac{{random\_draw\left( {N\left( {0,1} \right)} \right)}}{{\surd {\Delta t}}}$ before adding it to deterministic part. The noise term dynamic is like random walk, and the system is considered with no memory^11,22,34,35,54^.

In this study, Poisson process is employed to model the noise to formulate SPDE for transport of antibiotic and its resistant culture in the aquatic environment of the river. Poisson distribution could be employed in the processes where: a definite number of times an event occurs; the occurrence of one event is independent of the other; the average rate of the event is also independent of other the occurrences; and two events can not occur at the same time. Koyama et al.^24^ demonstrated that the frequency of cell counts of bacterial culture follows a Poisson distribution. Steven^45^ discussed how the random stochastic fluctuations in the microscopic processes follow a pattern of Poisson distribution and how it affects the patterns of nature in disease onset, rates of amino acids substitutions and composition of ecological communities. Mucha et al.^37^ presented a model for sedimentation of thin cells where velocity fluctuations are predicted by independent-Poisson-distribution estimates. Radioactive decay rates are reported to follow the Poisson distribution^6^. In ecological theory and practices, Poisson distribution usually represents a baseline against which other spatial patterns are compared, and its tractability helped in the development of the mathematics of population dynamics^47^.

The processes involved in the model are considered with a given number of events occurring in a fixed interval of time and/or space, these events occur with a known average rate and independently of the last time since the last event. Hence, the processes in the model possess the characteristics which make them appropriate to employ Poisson distribution for diffusion approximation. Another reason to employ Poisson distribution is that it has the unusual characteristics that both mean and variance are given as the same value which makes the calculations simple (Volkova et al., 2 013). For example, an event such as losing the plasmid carrying resistance gene (segregation) at a small Δt, is expected to be a rare random event in the population of bacteria. Hence, bacteria segregated at Δt are assumed to follow a Poisson distribution, and the variance in the number of loss equaled the mean.

### Sensitivity analysis

Sensitivity analysis is employed: to analyze the variations in the results due to changes in model parameters and; in determining the most influential parameters which can affect the accuracy of model results. It is very difficult to accurately represent an aquatic environment mathematically due to complex, random, and nonlinear processes involved in it. Uncertainties from measured data, model formulations, and model parameters affect the accuracy of model output. Hence, with sensitivity analysis tool the relationship between uncertainty in parameter values and model results can be clarified. Sensitivity analysis is an important tool to examine the changes in model results quantitatively concerning changes in model parameters. In present study, sensitivity analysis is performed by evaluating the effects on model results by changing one model parameter at a time^16,41^. The model is sensitive to a parameter if the changes in that parameter causes a large change in the model results. In this study, sensitivity analysis of the model is investigated for the variations in the concentration of plasmid-mediated resistant culture (${\text{n}}_{{{\text{psed}}}} ){ }$ in the sediment of the river. Hence, the model is simulated for a minimum and maximum value of each parameter given in Table 1. The concentration of plasmid-mediated resistant culture in sediments is observed after simulation run, and the relative difference in end results is the basis for the selection of sensitive parameters. Following are the parameters which are found to be sensitive in the analysis: adsorption rate of antibiotic with DOM, adsorption rate of antibiotic with TSS, settling rate, antibiotic degradation rate, , death rate, half rate constant, MIC, maximum growth rate in the water column, segregation rate, yield coefficient of susceptible bacteria, horizontal rate of conjugation, initial boundary concentrations of antibiotic and dissolved organic matter.

### Stochastic partial differential equations

Sources of stochasticity in the model are categorized into three parts:

- Demographic noise model formulation: Here we assumed that main source of stochasticity in the population of the resistant culture is due to variation in biological components such as growth rate, substrate utilization rate, the half rate constant, MIC, deaths, segregation, the rate of horizontal transfer of plasmid in bacteria.
- Environmental noise model formulation: The main source of stochasticity in the population of resistant bacteria is due to fluctuations in environmental parameters such as settling rate, Solids partition coefficient with antibiotics, DOM partition constant with antibiotics, degradation of antibiotics.
- Anthropogenic (upstream boundary condition) noise model formulation: The source of stochasticity in the population of resistance culture is due to fluctuations in the concentration of upstream boundary values such as antibiotic and dissolved organic matter.

The standard deviation of the process calculated from the deterministic rate at Δt is the average stochastic variation, i.e., √ (*deterministic rate).* Similarly, stochasticity is applied in the other processes rates. Randomness in individual parameter is represented with the stochastic part which is drawn ($\xi_{i}$) independently for each parameter^4,25,27,30,53^. We assumed the same magnitude of stochasticity for all the parameters in this model. The parameters which are employed for demographic stochasticity are death rate, half rate constant, MIC, maximum growth rate in the water column, segregation rate, yield coefficient of susceptible bacteria, and horizontal rate of conjugation. The parameters which are employed for environmental stochasticity are adsorption rate of antibiotic with DOM, adsorption rate of antibiotic with TSS, settling rate, antibiotic degradation rate. The parameters which are employed for anthropogenic stochasticity are concentration of antibiotic and concentration of dissolved organic matter.

The general form of SPDE for the concentration of resistant culture in aquatic environment ($n_{pwC}$) is presented below. The average stochastic fluctuations due to settling rate, death rate of bacteria, horizontal rate of conjugation, and, segregation rate is added to its deterministic partial differential equations.

$$ \begin{aligned} \frac{{\partial n_{pwC} }}{\partial t} & = \overbrace {{D\frac{{\partial^{2} n_{pwC} }}{{\partial x^{2} }} - u\frac{{\partial n_{pwC} }}{\partial x} - \left\{ {k_{set} n_{pwC} } \right\} + \frac{{k_{resus} }}{\gamma }n_{psed} }}^{Deterministic\;transport} \\ & \quad + \overbrace {{\left( {\left( {1 - \frac{{N_{wC} }}{{N_{wcmax} }}} \right)n_{pwC} \cdot \mu_{pwC} } \right)}}^{Deterministic\;bacterial\;growth} - \underbrace {{\left\{ {a \cdot n_{pwC} } \right\}}}_{Deterministic\;bacterial\;growth} + \overbrace {{\left\{ {\beta \frac{{n_{fwC} n_{pwC} }}{{n_{fwC} + n_{pwC} }}} \right\} + \left\{ {\beta \frac{{n_{fwC} n_{cpwC} }}{{n_{fwC} + n_{cpwC} }}} \right\}}}^{Deterministic\;plasmid\;transfer} - \underbrace {{\left\{ {SA \cdot n_{pwC} } \right\}}}_{Deterministic\;segregation} \\ & \quad - \left\{ {\underbrace {{\left( {\sqrt {k_{set} n_{pwC} } } \right)}}_{stochastic\;settling}\overbrace {{\xi_{i} }}^{Noise\;term\;(random\;process)}} \right\} - \left\{ {\underbrace {{\left( {\sqrt {a \cdot n_{pwC} } } \right)}}_{Stochastic\;bacterial\;death}\overbrace {{\xi_{i} }}^{Noise\;term}} \right\} + \underbrace {{\left\{ {\left( {\sqrt {\beta \frac{{n_{fwC} n_{pwC} }}{{n_{fwC} + n_{pwC} }}} } \right)\overbrace {{\xi_{i} }}^{Noise\;term}} \right\} + \left\{ {\left( {\beta \frac{{n_{fwC} n_{cpwC} }}{{n_{fwC} + n_{cpwC} }}} \right)\overbrace {{\xi_{i} }}^{Noise\;term}} \right\}}}_{Stochastic\;plasmid\;transfer} - \left\{ {\underbrace {{\left( {\sqrt {SA \cdot n_{pwC} } } \right)}}_{Stochastic\;segregation}\overbrace {{\xi_{i} }}^{Noise\;term}} \right\} \\ \end{aligned} $$

The SPDEs for the change in concentrations of fluoroquinolone (A), heavy metal (M), total suspended solid (TSS), particulate organic matter (POM), dissolved organic matter (DOM), population of susceptible bacteria (${\text{n}}_{f}$) and population of resistant bacteria (${\text{n}}_{f} ,{\text{ n}}_{p} ,{\text{ n}}_{c} ,{\text{n}}_{cp}$) in aquatic environment (wc) and sediment bed (sed) are presented without deterministic terms to simplify the model for clarity. Full equations are mentioned in supplementary information.

- Fluoroquinolone
  The Eqs. (1) and (2) represent the concentration of antibiotic in water column and sediment respectively, where settling rate and antibiotic degradation rate parameters are making their respective process stochastic.
  1 $$ \frac{{\partial A_{wC} }}{\partial t} = - \left\{ {\left. { \left( {\sqrt {k_{set} A_{pwc} } } \right)\xi } \right\}} \right. + - \left\{ {\left. {\left( {\sqrt { K_{xawc } A_{wC} } } \right)\xi } \right\}} \right. $$
  2 $$ \frac{{\partial A_{sed} }}{\partial t} = \left\{ {\left. {\left( {k_{set} \gamma A_{pwc} } \right) + \left( {\sqrt {k_{set} \gamma A_{pwc} } } \right)\xi } \right\}} \right. $$
- Metals
  Equations (3) and (4) represent the metal concentration in the river where settling rate and yield coefficient of susceptible bacteria are considered for stochasticity.
  3 $$ \frac{{\partial M_{sed} }}{\partial t} = \left\{ {\left. {\left( {\sqrt {k_{set} \gamma M_{pwc} } } \right)\xi } \right\}} \right. - \eta \left( {\left\{ {\left. {\sqrt {\frac{{\mu_{f,sed } n_{f,sed} }}{{Y_{f} }}} \xi } \right\}} \right.} \right) $$
  4 $$ \frac{{\partial M_{wC} }}{\partial t} = - \left\{ {\left. {\left( {\sqrt {k_{set} M_{pwc} } } \right)\xi } \right\}} \right. - \eta \left( {\left\{ {\left. {\sqrt {\frac{{\mu_{f,wc } n_{f,wc} }}{{Y_{f} }}} \xi } \right\}} \right.} \right) $$
- Total suspended solids
  Similarly, Eqs. (5) and (6) are representing the concentration of TSS in the river where the only parameter which is stochastic is the settling rate.
  5 $$ \frac{{\partial TSS_{wC} }}{\partial t} = - \left\{ {\left. {\left( {\sqrt {k_{Set} TSS_{wC} } } \right)\xi } \right\}} \right. $$
  6 $$ \frac{{\partial TSS_{sed} }}{\partial t} = \left\{ {\left. {\left( {\sqrt {k_{Set} \gamma TSS_{wC} } } \right)\xi } \right\}} \right. $$
- Particulate organic matter
  The concentration of particulate organic matter in the river is represented by Eqs. (7) and (8), the parameters employed for stochasticity in these equations are settling rate and death rate of bacteria.
  7 $$ \frac{{\partial \left( {POM_{wc} } \right)}}{\partial t} = \left\{ {\left. {\left( {\sqrt {k_{set} POM_{wc} } } \right)\xi } \right\}} \right. + \left\{ {\left. {\left( {\sqrt {a\left( {n_{fwC} + n_{pwC} + n_{cwC} + n_{cpwC} } \right)} } \right) \xi } \right\}} \right. $$
  8 $$ \frac{{\partial \left( {POM_{sed} } \right)}}{\partial t} = \left\{ {\left. {\left( {\sqrt {k_{set} \gamma POM_{wc} } } \right)\xi } \right\}} \right. + \left\{ {\left. {\left( {\sqrt {a\left( {n_{fsed} + n_{psed} + n_{csed} + n_{cpsed} } \right)} } \right)\xi } \right\}} \right. $$
- Dissolved organic matter
  Equations (9) and (10) are representing the concentration of DOM in the river, the stochasticity is only due to yield coefficient of susceptible bacteria.
  9 $$ \frac{{\partial \left( {DOM_{wc} } \right)}}{\partial t} = - \left( {\sqrt {\frac{{\mu_{f,wc } n_{f,wc} }}{{Y_{f} }}} \xi } \right) $$
  10 $$ \frac{{\partial \left( {DOM_{sed} } \right)}}{\partial t} = - \left( {\sqrt {\frac{{\mu_{f,sed } n_{f,sed} }}{{Y_{f} }}} \xi } \right) $$
- Susceptible and resistant bacteria
  Equations from (11) to (17) are representing mass balance equation for the concentration of susceptible and resistant bacteria in the water column and sediment bed. The stochasticity due to settling rate, death rate of bacteria, horizontal rate of conjugation, and, segregation rate is added to the deterministic partial differential equations.
  11 $$ \frac{{\partial n_{fwC} }}{\partial t} = - \left\{ {\left. {\left( {\sqrt {k_{set} n_{fwC} } } \right)\xi } \right\}} \right. - \left\{ {\left. { \left( {\sqrt {a.n_{fwC} } } \right)\xi } \right\}} \right. - \left\{ {\left. { \left( {\sqrt {\beta \frac{{n_{fwC} n_{pwC} }}{{n_{fwC} + n_{pwC} }}} } \right)\xi } \right\}} \right. - \left\{ {\left. { \left( {\sqrt {\beta \frac{{n_{fwC} n_{cpwc} }}{{n_{fwC} + n_{cpwC} }}} } \right)\xi } \right\}} \right. + \left\{ {\left. { \left( {\sqrt {SA.n_{pwC} } } \right)\xi } \right\}} \right. $$
  12 $$ \frac{{\partial n_{fsed} }}{\partial t} = \left\{ {\left. {\left( {\sqrt {k_{set} \gamma n_{fsed} } } \right)\xi } \right\}} \right. - \left\{ {\left. {\left( {\sqrt {a.n_{fsed} } } \right)\xi } \right\}} \right. - \left\{ {\left. { \left( {\sqrt {\beta \frac{{n_{fsed} n_{psed} }}{{n_{fsed} + n_{psed} }}} } \right)\xi } \right\} - } \right.\left\{ {\left. { \left( {\sqrt {\beta \frac{{n_{fsed} n_{cpsed} }}{{n_{fsed} + n_{cpsed} }}} } \right)\xi } \right\}} \right. + \left\{ {\left. {\left( {\sqrt {SA.n_{psed} } } \right)\xi } \right\}} \right. $$
  13 $$ \frac{{\partial n_{pwC} }}{\partial t} = \left\{ {\left. { \left( {\sqrt {k_{set} n_{pwC} } } \right)\xi } \right\}} \right. + - \left\{ {\left. { \left( {\sqrt {a.n_{pwC} } } \right)\xi } \right\}} \right. + \left\{ {\left. { \left( {\sqrt {\beta \frac{{n_{fwC} n_{pwC} }}{{n_{fwC} + n_{pwC} }}} } \right)\xi } \right\} + } \right.\left\{ {\left. { \left( {\sqrt {\beta \frac{{n_{fwC} n_{cpwc} }}{{n_{fwC} + n_{cpwC} }}} } \right)\xi } \right\}} \right. - \left\{ {\left. { \left( {\sqrt {SA.n_{pwC} } } \right)\xi } \right\}} \right. $$
  14 $$ \frac{{\partial n_{psed} }}{\partial t} = \left\{ {\left. { \left( {\sqrt {k_{set} \gamma n_{psed} } } \right)\xi } \right\}} \right. - \left\{ {\left. { \left( {\sqrt {a.n_{psed} } } \right)\xi } \right\}} \right. + \left\{ {\left. {\left( {\sqrt {\beta \frac{{n_{fsed} n_{psed} }}{{n_{fsed} + n_{psed} }}} } \right)\xi } \right\}} \right. + \left\{ {\left. { \left( {\sqrt {\beta \frac{{n_{fsed} n_{cpsed} }}{{n_{fsed} + n_{cpsed} }}} } \right)\xi } \right\} - \left\{ {\left. {\left( {\sqrt {SA.n_{psed} } } \right)\xi } \right\}} \right.} \right. $$
  15 $$ \frac{{\partial n_{cpwc} }}{\partial t} = - \left\{ {\left. {\left( {\sqrt {k_{set} n_{cpwC} } } \right)\xi } \right\}} \right. - \left\{ {\left. {\left( {\sqrt {a.n_{cpwC} } } \right)\xi } \right\}} \right. + \left\{ {\left. {\left( {\sqrt {\beta \frac{{n_{cpwC} n_{cwC} }}{{n_{cpwC} + n_{cwC} }}} } \right)\xi } \right\} + \left\{ {\left. {\left( {\sqrt {\beta \frac{{n_{cwC} n_{pwc} }}{{n_{cwC} + n_{pwC} }}} } \right)\xi } \right\}} \right.} \right. - \left\{ {\left. {\left( {\sqrt {SA.n_{cpwC} } } \right)\xi } \right\}} \right. $$
  16 $$ \frac{{\partial n_{cpsed} }}{\partial t} = \left\{ {\left. {\left( {\sqrt {k_{set} \gamma n_{cpsed} } } \right)\xi } \right\}} \right. + - \left\{ {\left. {\left( {\sqrt {a.n_{cpsed} } } \right)\xi } \right\}} \right. + \left\{ {\left. {\left( {\sqrt {\beta \frac{{n_{cpsed} n_{csed} }}{{n_{cpsed} + n_{csed} }}} } \right)\xi } \right\} + \left\{ {\left. { \left( {\sqrt {\beta \frac{{n_{csed} n_{psed} }}{{n_{csed} + n_{psed} }}} } \right)\xi } \right\}} \right.} \right. - \left\{ {\left. {\left( {\sqrt {SA.n_{cpsed} } } \right)\xi } \right\}} \right. $$
  17 $$ \frac{{\partial n_{cwC} }}{\partial t} = - \left\{ {\left. { \left( {\sqrt {k_{set} n_{cwC} } } \right)\xi } \right\}} \right. + - \left\{ {\left. {\left( {\sqrt {a.n_{cwC} } } \right)\xi } \right\}} \right. - \left\{ {\left. {\left( {\sqrt {\beta \frac{{n_{cwC} n_{pwC} }}{{n_{cwC} + n_{pwC} }}} } \right)\xi } \right\}} \right. - \left\{ {\left. {\left( {\sqrt { \beta \frac{{n_{cwC} n_{cpwc} }}{{n_{cwC} + n_{cpwC} }}} } \right)\xi } \right\}} \right. + \left\{ {\left. {\left( {\sqrt {SA.n_{cpwC} } } \right)\xi } \right\}} \right. $$

## Results and discussion

### Sensitivity analysis

**Figure 2:**
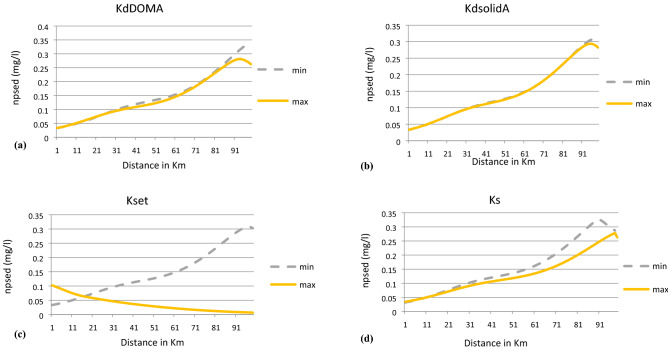
(**a**) Sensitive analysis of DOM partition constant with antibiotics (${\text{K}}_{dDOMA}$), (**b**) sensitive analysis of solids partition coefficient with antibiotics (${\text{K}}_{dsolidA}$), (**c**) sensitive analysis of setting rate constant (${\text{K}}_{{{\text{set}}}}$), (**d**) sensitive analysis of half rate constant (${\text{K}}_{s}$), (**e**) sensitive analysis of death rate constant (a), (**f**) sensitive analysis of constant for degradation of antibiotics in water column (${\text{K}}_{xawc}$), (**g**) sensitive analysis of minimum inhibitory concentration (MIC), (**h**) sensitive analysis of maximum specific growth rate in water column ($\upmu _{{{\text{maxwc}}}}$). (**i**) sensitive analysis of rate of segregatin (SA). (**j**) sensitive analysis of yield coefficient of susceptible bacteria (${\text{Y}}_{f}$).

Solids partition coefficient with antibiotics (${\text{K}}_{dsolidA}$) was found to be least sensitive. The maximum difference in the output results was 0.05 mg/l. Hence, ${\text{K}}_{dsolidA}$ was considered as the cut off value to qualify a parameter to be sensitive. The maximum difference in the output results for other parameters such as: DOM partition coefficient with antibiotics (${\text{K}}_{dDOMA}$), setting rate (${\text{K}}_{{{\text{set}}}}$), half rate constant (${\text{K}}_{s}$), yield coefficient of susceptible bacteria (${\text{Y}}_{f}$), death rate (a), constant for degradation of antibiotics in water column (${\text{K}}_{xawc}$), minimum inhibitory concentration (MIC) , maximum specific growth rate in water column (${\upmu }_{{{\text{maxwc}}}}$), rate of segregation (SA), and rate of horizontal transfer of plasmid (β) was observed to be 0.08, 0.3 mg/l, 0.09 mg/l, 0.12 mg/l, 0.75 mg/l, 0.22 mg/l, 0.16 mg/l, 0.41 mg/l, 0.15 mg/l and 0.7 mg/l respectively. Remaining other parameters were not found to be sensitive at all. Hence, stochasticity only for those parameters was included in the formulation of SPDEs which were considered sensitive. The graphs for simulation end results for model sensitive analysis are mentioned in Fig. 2(a–j).

### SPDE simulation results

**Figure 3:**
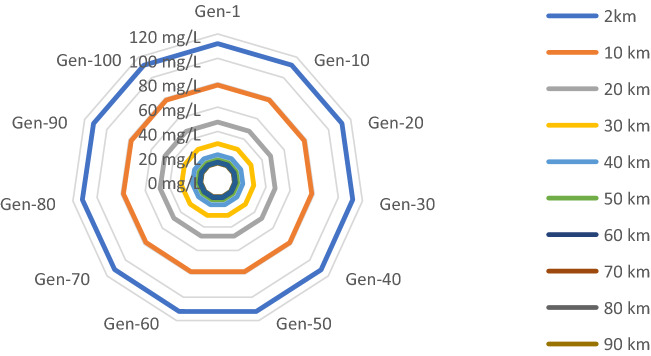
Variation in concentration of npsed (mg/l) due to demographic stochasticity w.r.t generations at different distances in river sediment.

The SPDE model is simulated for demographic stochasticity, environmental stochasticity, and anthropogenic stochasticity. The model is simulated for 100 generations, and the time in each generation is till the state variables reach steady state condition. With all the stochastic formulations the plasmid-mediated resistant bacteria population reached its stochastic equilibrium within 5 years. The variation in the concentration of ${\text{n}}_{{{\text{psed}}}}$ due to demographic, environmental and anthropogenic stochasticity for 100 generations at different distances in river sediment was observed not to be significant. The concentration of ${\text{n}}_{{{\text{psed}}}} { }$ is varying in small ranges of multiples of μg/l (standard deviation minimum: 2.672 X10^-6 and standard deviation maximum: 0.0982) with respect to the large x-axis values (time or distance). There was no distinguished difference in the plots of ${\text{n}}_{{{\text{psed}}}}$ due to demographic, environmental and anthropogenic stochasticity. Hence, only one plot is presented as Fig. 3 to show the variations in concentration of ${\text{n}}_{{{\text{psed}}}}$ (mg/l) due to demographic stochasticity w.r.t generations at different distances in river sediment. Colored lines in the radar plot represent the resistant bacterial population at a distance point in the river, and the vertices represent the time in terms of generations. The variation in the population of ${\text{n}}_{{{\text{psed}}}}$ is shown by the colored lines at different radial distances, data axis is shown across the radar chart. The ${\text{n}}_{{{\text{psed}}}}$ value is reaching a steady state in one generation time and afterwards it is not changing significantly, as shown in the plot, each colored line is almost at same radial distance from the center. However, the radial distances between the colored lines are varying. The colored line closest to the center is of highest ${\text{n}}_{{{\text{psed}}}}$ value which decreases with the increase in the radial distance. There is a large difference in the value of ${\text{n}}_{{{\text{psed}}}}$ at the 2 km and 10 km, however, difference decreases between the value of ${\text{n}}_{{{\text{psed}}}}$ as we move out towards the circumference, which means that the resistant bacterial population is reaching a steady state condition at 100 km distance point in the river.

**Figure 4:**
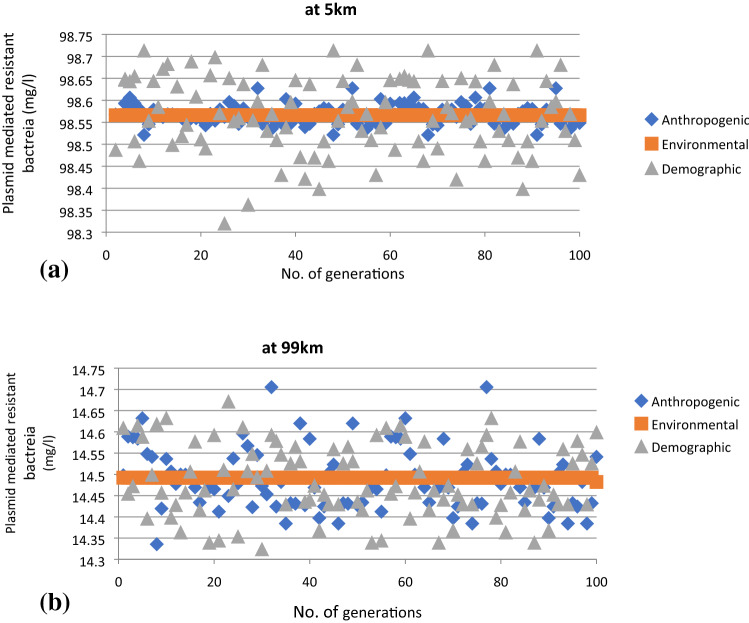
(**a**) Variations in population of plasmid associated resistant bacteria population in sediment ($n_{psed}$) at 5 km distance from starting point. (**b**) Variations in population of plasmid associated resistant bacteria population in sediment ($n_{psed}$) at 5 km and 99 km distance from starting point.

The graphs are plotted at 5 km and 99 km Fig. 4a,b to show the comparison of the obtained distribution of antibiotic resistant bacteria due to stochasticity. The distribution of resistant bacteria differed depending on the assumptions made about the main source of stochasticity in dynamics of antibiotic resistance. The distribution outcome of resistant bacteria population was observed to be negligible (standard deviation 7.225X10^-6 at 5 km and 0.00099 at 99 km, Fig. 4a,b and mean close to that from the deterministic model when stochasticity was applied to environmental parameters of the model. The distribution of population of resistant bacteria due to stochasticity in demographic parameters of the model was also observed to be small but more uniform and evident in the entire stretch of the river, (standard deviation 0.087 at 5 km and 0.084 at 99 km). However, the distribution of resistant population due to stochasticity in anthropogenic parameters of the model was observed to be narrower in the initial stretch of the river and became more distributed in the downstream (standard deviation 0.023 at 5 km and 0.071 at 99 km).

The inference can be made with this study that the application of stochasticity in the parameters of the one-dimensional steady state transport model poses insignificant effects on the population of resistant bacteria in sediments. When entire system reaches a steady there is no impact on the resistant bacterial population due to randomness in the variables which play role in the dynamics of resistant population.

## Conclusion

Stochastic effect of model parameters on the population of resistant bacteria in an antibiotic polluted river was examined in this study. The sensitive analyses were performed to find out the most influential variables and parameters which could affect the preciseness of model output. We categorized those possible sources of stochasticity in the demographic, environmental and anthropogenic groups to evaluate the influence of variations in the parameters over the population of resistant bacteria in sediment. Stochasticity due to demographic parameters and anthropogenic (u/s boundary condition) variables were found not to be significantly influencing the amount of resistance (only in multiples of μg/l). Stochasticity in environmental parameters was not at all influencing the population of resistant culture in the sediments.

Musi river is polluted with high concentrations of antibiotics and there is an urgent need to perform a risk assessment of the nearby aeras which are impacted by its water. Hence developing a refined model for the transport of antibiotics and transport of antibiotic resistant bacteria in the polluted river is a necessity. A robust model is required for precise temporal-spatial prediction of resistant bacteria. Two-dimensional and three-dimensional stochastic transport model be could be developed to obtain high accuracy results. However, for a better understanding of the behavior of stochasticity in the system one-dimensional model can be used, and with more field data for a sophisticated validation. The availability of site-specific data can facilitate an improved calibration and verification of model, which could play a huge role in making the model robust and successful. There is a substantial gap between the theory of stochastic resistant bacterial growth dynamics and the theory of transport of antibiotic resistance in polluted aquatic environment, this study is an effort towards bridging this gap.

## Supplementary information

Supplementary information.
