# Supplementary material for: Modeling transport of antibiotic resistant bacteria in aquatic environment using stochastic differential equations
Source: Sci Rep. 2020 Sep 15;10:15081. doi: 10.1038/s41598-020-72106-3 (PMC7494867; doi:10.1038/s41598-020-72106-3)
Supplement: Supplementary file 1 — Supplementary information. [file 41598_2020_72106_MOESM1_ESM.pdf]

Modeling transport of antibiotic resistant bacteria in aquatic environment using stochastic differential equations

Ritu Gothwal

Research Scholar, Department of Civil Engineering, Indian Institute of Technology Hyderabad, Kandi Sangareddy 502285, Telangana, India

Email: gothwalritu [at] gmail.com

Shashidhar Thatikonda*

Faculty, Department of Civil Engineering, Indian Institute of Technology Hyderabad, Kandi Sangareddy 502285, Telangana, India

* Corresponding author: S. Thatikonda, Department of Civil Engineering, Indian Institute of Technology Hyderabad, Kandi, Sangareddy, Telangana 502285, India. Phone: +91 40 2301-6107; +91 8096 442220; Fax: (040) 2301 6032. Email: shashidhar [at] iith.ac.in

S.1. Stochastic differential equations:

S.1.1. Fluoroquinolone partitioning in water and sediment column

$$A_{fd,wc} = \left(\frac{A_{wC}}{1 + K_{d,DOM,A}DOM_{wC} + K_{d,Solid,A}TSS_{wC}}\right) + \left(\sqrt{\frac{A_{wC}}{1 + K_{d,DOM,A}DOM_{wC} + K_{d,Solid,A}TSS_{wC}}}\right)\xi \quad (1)$$

$$A_{fd,sed} = \left(\frac{A_{sed} \cdot ø}{ø + K_{d,DOM,A}DOM_{sed} + K_{d,Solid,A}TSS_{sed}}\right) + \left(\sqrt{\frac{A_{sed} \cdot ø}{ø + K_{d,DOM,A}DOM_{sed} + K_{d,Solid,A}TSS_{sed}}}\right)\xi \quad (2)$$

$$A_{d,wc} = \left(\frac{A_{wC}(K_{d,DOM,A}DOM_{wC})}{1 + K_{d,DOM,A}DOM_{wC} + K_{d,Solid,A}TSS_{wC}}\right) + \left(\sqrt{\frac{A_{wC}(K_{d,DOM,A}DOM_{wC})}{1 + K_{d,DOM,A}DOM_{wC} + K_{d,Solid,A}TSS_{wC}}}\right)\xi \quad (3)$$

$$A_{d,sed} = \left(\frac{A_{sed}(K_{d,DOM,A}DOM_{sed})}{ø + K_{d,DOM,A}DOM_{sed} + K_{d,Solid,A}TSS_{sed}}\right) + \left(\sqrt{\frac{A_{sed}(K_{d,DOM,A}DOM_{sed})}{ø + K_{d,DOM,A}DOM_{sed} + K_{d,Solid,A}TSS_{sed}}}\right)\xi \quad (4)$$

$$A_{p,wc} = \left(\frac{A_{wC}\, K_{d,Solid,A}TSS_{wC}}{1 + K_{d,DOM,A}DOM_{wC} + K_{d,Solid,A}TSS_{wC}}\right) + \left(\sqrt{\frac{A_{wC}\, K_{d,Solid,A}TSS_{wC}}{1 + K_{d,DOM,A}DOM_{wC} + K_{d,Solid,A}TSS_{wC}}}\right)\xi \quad (5)$$

$$A_{p,sed} = \left(\frac{A_{sed}\, K_{d,Solid,A}TSS_{sed}}{ø + K_{d,DOM,A}DOM_{sed} + K_{d,Solid,A}TSS_{sed}}\right) + \left(\sqrt{\frac{A_{sed}(ø + K_{d,DOM,A}DOM_{sed})}{ø + K_{d,DOM,A}DOM_{sed} + K_{d,Solid,A}TSS_{sed}}}\right)\xi \quad (6)$$

S.1.2 Metal partitioning in water and sediment column

$$M_{fd,wc} = \frac{M_{wC}}{1 + K_{d,DOM,M} DOM_{wC} + K_{d,Solid,M} TSS_{wC}} \quad (7)$$

$$M_{fd,sed} = \frac{M_{sed}.\ ø}{ø + K_{d,DOM,M} DOM_{sed} + K_{d,Solid,M} TSS_{sed}} \quad (8)$$

$$M_{d,wc} = \frac{M_{wC}(K_{d,DOM,M} DOM_{wC})}{1 + K_{d,DOM,M} DOM_{wC} + K_{d,Solid,M} TSS_{wC}} \quad (9)$$

$$M_{d,sed} = \frac{M_{sed}(K_{d,DOM,M} DOM_{sed})}{ø + K_{d,DOM,M} DOM_{sed} + K_{d,Solid,M} TSS_{sed}} \quad (10)$$

$$M_{p,wc} = \frac{M_{wC}\ K_{d,Solid,M} TSS_{wC}}{1 + K_{d,DOM,M} DOM_{wC} + K_{d,Solid,M} TSS_{wC}} \quad (11)$$

$$M_{p,sed} = \frac{M_{sed}\ K_{d,Solid,M} TSS_{sed}}{ø + K_{d,DOM,M} DOM_{sed} + K_{d,Solid,M} TSS_{sed}} \quad (12)$$

S.1.3 Bacterial growth

$$\mu_{f,wc} = \begin{cases} \mu_{maxwc} \left(\frac{k_i}{k_i + M_{fd,wc}}\right)\left(1 - \frac{A_{fd,wc}}{MIC}\right)\left(\frac{DOM_{wc}}{K_s + DOM_{wc}}\right) + \left(\sqrt{\mu_{maxwc} \left(\frac{k_i}{k_i + M_{fd,wc}}\right)\left(1 - \frac{A_{fd,wc}}{MIC}\right)\left(\frac{DOM_{wc}}{K_s + DOM_{wc}}\right)}\right)\ \xi, & A_{fd} < MIC \\ 0, & A_{fd} \geq \text{MIC} \end{cases} \quad (13)$$

$$\mu_{f,sed} = \begin{cases} \mu_{maxsed}\left(\frac{k_i}{k_i + M_{fd,sed}}\right)\left(1 - \frac{A_{fd,sed}}{MIC}\right)\left(\frac{DOM_{sed}}{K_s + DOM_{sed}}\right) + \sqrt{\mu_{maxsed}\left(\frac{k_i}{k_i + M_{fd,sed}}\right)\left(1 - \frac{A_{fd,sed}}{MIC}\right)\left(\frac{DOM_{sed}}{K_s + DOM_{sed}}\right)}\ \xi, A_{fd} < MIC \\ 0, \quad A_{fd} \geq \mathrm{MIC} \end{cases} \tag{14}$$

$$\mu_{p,wc} = \left\{\mu_{maxwc}\left(\frac{DOM_{wc}}{K_s+DOM_{wc}}\right)\left(\frac{k_i}{k_i+M_{fd,wc}}\right)\left(1 - C_p - X\right)\left(\frac{MIC}{MIC+A_{fd,wc}}\right)\right\} + \left\{\sqrt{\mu_{maxwc}\left(\frac{DOM_{wc}}{K_s+DOM_{wc}}\right)\left(\frac{k_i}{k_i+M_{fd,wc}}\right)\left(1 - C_p - X\right)\left(\frac{MIC}{MIC+A_{fd,wc}}\right)}\ \xi\right\} \tag{15}$$

$$\mu_{p,sed} = \left\{\mu_{maxsed}\left(\frac{DOM_{sed}}{K_s+DOM_{sed}}\right)\left(\frac{k_i}{k_i+M_{fd,sed}}\right)\left(1 - C_p - X\right)\left(\frac{MIC}{MIC+A_{fd,sed}}\right)\right\} + \left\{\sqrt{\mu_{maxsed}\left(\frac{DOM_{sed}}{K_s+DOM_{sed}}\right)\left(\frac{k_i}{k_i+M_{fd,sed}}\right)\left(1 - C_p - X\right)\left(\frac{MIC}{MIC+A_{fd,sed}}\right)}\xi\right\} \tag{16}$$

$$\mu_{c,wc} = \left\{\mu_{maxwc}\left(\frac{DOM_{wc}}{K_s+DOM_{wc}}\right)\left(\frac{k_i}{k_i+M_{fd,wc}}\right)\left(1 - C_c\right)\left(\frac{MIC}{MIC+A_{fd,wc}}\right)\right\} + \left\{\sqrt{\mu_{maxwc}\left(\frac{DOM_{wc}}{K_s+DOM_{wc}}\right)\left(\frac{k_i}{k_i+M_{fd,wc}}\right)\left(1 - C_c\right)\left(\frac{MIC}{MIC+A_{fd,wc}}\right)}\ \xi\right\} \tag{17}$$

$$\mu_{c,sed} = \left\{\mu_{maxsed}\left(\frac{DOM_{sed}}{K_s+DOM_{sed}}\right)\left(\frac{k_i}{k_i+M_{fd,sed}}\right)\left(1 - C_c\right)\left(\frac{MIC}{MIC+A_{fd,sed}}\right)\right\} + \left\{\sqrt{\mu_{maxsed}\left(\frac{DOM_{sed}}{K_s+DOM_{sed}}\right)\left(\frac{k_i}{k_i+M_{fd,sed}}\right)\left(1 - C_c\right)\left(\frac{MIC}{MIC+A_{fd,sed}}\right)}\ \xi\right\} \tag{18}$$

$$\mu_{cp,wc} = \mu_{maxwc}\left\{\left(\frac{DOM_{wc}}{K_s+DOM_{wc}}\right)\left(\frac{k_i}{k_i+M_{fd,wc}}\right)\left(1 - C_c - C_p - X\right)\left(\frac{MIC}{MIC+A_{fd,wc}}\right)\right\} + \left\{\sqrt{\mu_{maxwc}\left(\frac{DOM_{wc}}{K_s+DOM_{wc}}\right)\left(\frac{k_i}{k_i+M_{fd,wc}}\right)\left(1 - C_c - C_p - X\right)\left(\frac{MIC}{MIC+A_{fd,wc}}\right)}\ \xi\right\} \tag{19}$$

$$\mu_{cp,sed} = \mu_{maxsed} \left\{\left(\frac{DOM_{sed}}{K_s+DOM_{sed}}\right)\left(\frac{k_i}{k_i+M_{fd,sed}}\right)(1-C_c-C_p-X)\left(\frac{MIC}{MIC+A_{fd,sed}}\right)\right\} + \left\{\sqrt{\mu_{maxsed} \left(\frac{DOM_{sed}}{K_s+DOM_{sed}}\right)\left(\frac{k_i}{k_i+M_{fd,sed}}\right)(1-C_c-C_p-X)\left(\frac{MIC}{MIC+A_{fd,sed}}\right)}\,\xi\right\} \quad (20)$$

S.1.4 Mass balance equations

a) Fluoroquinolones

$$\frac{\partial A_{wC}}{\partial t} = D\frac{\partial^2 A_{wC}}{\partial x^2} - u\frac{\partial A_{wC}}{\partial dx} - \left\{(k_{set}A_{pwc}) + \left(\sqrt{k_{set}A_{pwc}}\right)\xi\right\} + \frac{k_{resus}}{\gamma}A_{sed} - \frac{k_{diff}}{h}\left(A_{dwc} + A_{fd,wc} - \frac{A_{d,sed}}{ø} - \frac{A_{fd,sed}}{ø}\right) - \{(K_{xawc}A_{wC}) + (\sqrt{K_{xawc}A_{wC}})\xi\} \quad (21)$$

$$\frac{\partial A_{sed}}{\partial t} = \left\{(k_{set}\gamma A_{pwc}) + \left(\sqrt{k_{set}\gamma A_{pwc}}\right)\xi\right\} - k_{resus}A_{sed} - \frac{k_{diff}}{d}\left(\frac{A_{d,sed}}{ø} + \frac{A_{fd,sed}}{ø} - A_{d,wc} - A_{fd,wc}\right) - K_{xased}A_{sed} \quad (22)$$

b) Metals

$$\frac{\partial M_{wC}}{\partial t} = D\frac{\partial^2 M_{wC}}{\partial x^2} - u\frac{\partial M_{wC}}{\partial x} - \left\{(k_{set}M_{pwc}) + \left(\sqrt{k_{set}M_{pwc}}\right)\xi\right\} + \frac{k_{resus}}{\gamma}M_{sed} - \frac{k_{diff}}{h}\left(M_{d,wc} + M_{fd,wc} - \frac{M_{d,sed}}{ø} - \frac{M_{fd,sed}}{ø}\right) - \eta\left(\left\{\frac{\mu_{f,wc}n_{f,wc}}{Y_f} + \left(\sqrt{\frac{\mu_{f,wc}n_{f,wc}}{Y_f}}\,\xi\right)\right\} + \frac{\mu_{p,wc}n_{p,wc}}{Y_p} + \frac{\mu_{c,wc}n_{c,wc}}{Y_c} + \frac{\mu_{cp,wc}n_{cp,wc}}{Y_{cp}}\right) \quad (23)$$

$$\frac{\partial M_{sed}}{\partial t} = \left\{k_{set}\gamma M_{pwc} + \left(\sqrt{k_{set}\gamma M_{pwc}}\right)\xi\right\} - k_{resus}M_{sed} - \frac{k_{diff}}{d}\left(\frac{M_{d,sed}}{ø} + \frac{M_{fd,sed}}{ø} - M_{d,wc} - M_{fd,wc}\right) - \eta\left(\left\{\frac{\mu_{f,sed}n_{f,sed}}{Y_f} + \left(\sqrt{\frac{\mu_{f,sed}n_{f,sed}}{Y_f}}\,\xi\right)\right\} + \frac{\mu_{p,sed}n_{p,sed}}{Y_p} + \frac{\mu_{c,sed}n_{c,sed}}{Y_c} + \frac{\mu_{cp,sed}n_{cp,sed}}{Y_{cp}}\right) \quad (24)$$

c) Total suspended solids

$$\frac{\partial TSS_{wC}}{\partial t} = D\frac{\partial^2 TSS_{wC}}{\partial x^2} - u\frac{\partial TSS_{wC}}{\partial x} - \left\{(k_{Set}\, TSS_{wC}) + \left(\sqrt{k_{Set}\, TSS_{wC}}\right)\xi\right\} + \frac{k_{resus}}{\gamma} TSS_{sed} - Z\, K_{H,wc}\, TSS_{wC} \tag{25}$$

$$\frac{\partial TSS_{sed}}{\partial t} = \left\{(k_{Set}\gamma\, TSS_{wC}) + \left(\sqrt{k_{Set}\gamma\, TSS_{wC}}\right)\xi\right\} - k_{resus} TSS_{sed} - ZK_{H,sed}\, TSS_{sed} \tag{26}$$

d) Particulate organic matter

$$\frac{\partial (POM_{wc})}{\partial t} = D\frac{\partial^2 (POM_{wc})}{\partial x^2} - u\frac{\partial (POM_{wc})}{\partial x} - \left\{(k_{set} POM_{wc}) + \left(\sqrt{k_{set} POM_{wc}}\right)\xi\right\} + \frac{k_{resus}}{\gamma} POM_{sed} - K_{H,wc}\, POM_{wc} + \left\{a\left(n_{fwC} + n_{pwC} + n_{cwC} + n_{cpwC}\right) + \left(\sqrt{a\left(n_{fwC} + n_{pwC} + n_{cwC} + n_{cpwC}\right)}\right)\ \xi\right\} \tag{27}$$

$$\frac{\partial (POM_{sed})}{\partial t} = \left\{(k_{set}\gamma POM_{wc}) + \left(\sqrt{k_{set}\gamma POM_{wc}}\right)\xi\right\} - k_{resus} POM_{sed} - K_{H,sed}\, POM_{sed} + \left\{a\left(n_{fsed} + n_{psed} + n_{csed} + n_{cpsed}\right) + + \left(\sqrt{a\left(n_{fsed} + n_{psed} + n_{csed} + n_{cpsed}\right)}\right)\xi\right\} \tag{28}$$

e) Dissolved organic matter

$$\frac{\partial(DOM_{wc})}{\partial t} = D\frac{\partial^2(DOM_{wc})}{\partial x^2} - u\frac{\partial(DOM_{wc})}{\partial x} - \frac{k_{diff}}{h}\,(DOM_{wc} - DOM_{sed}) + \; K_{H,wc}\,POM_{wc} - \left(\left\{\frac{\mu_{f,wc}\,n_{f,wc}}{Y_f} + \left(\sqrt{\frac{\mu_{f,wc}\,n_{f,wc}}{Y_f}}\;\xi\right)\right\} + \frac{\mu_{p,wc}\,n_{p,wc}}{Y_p} + \frac{\mu_{c,wc}\,n_{c,wc}}{Y_c} + \frac{\mu_{cp,wc}\,n_{cp,wc}}{Y_{cp}}\right) \quad (29)$$

$$\frac{\partial(DOM_{sed})}{\partial t} = \frac{k_{diff}}{d}\,(DOM_{wc} - DOM_{sed}) + \; K_{H,sed}\,POM_{sed} - \left(\left\{\frac{\mu_{f,sed}\,n_{f,sed}}{Y_f} + \left(\sqrt{\frac{\mu_{f,sed}\,n_{f,sed}}{Y_f}}\,\xi\right)\right\} + \frac{\mu_{p,sed}\,n_{p,sed}}{Y_p} + \frac{\mu_{c,sed}\,n_{c,sed}}{Y_c} + \frac{\mu_{cp,sed}\,n_{cp,sed}}{Y_{cp}}\right) \quad (30)$$

f) Susceptible and resistant bacteria population

$$\frac{\partial n_{fwC}}{\partial t} = D\frac{\partial^2 n_{fwC}}{\partial x^2} - u\frac{\partial n_{fwC}}{\partial x} - \left\{k_{set}n_{fwC} + \left(\sqrt{k_{set}n_{fwC}}\right)\xi\right\} + \frac{k_{resus}}{\gamma}n_{fsed} + \left(\left(1 - \frac{N_{wC}}{N_{wcmax}}\right)n_{fwC}.\,\mu_{fwC}\right) - \left\{(a.\,n_{fwC}) + \left(\sqrt{a.\,n_{fwC}}\right)\xi\right\} - \left\{\beta\frac{n_{fwC}n_{pwC}}{n_{fwC}+n_{pwC}} + \left(\sqrt{\beta\frac{n_{fwC}n_{pwC}}{n_{fwC}+n_{pwC}}}\right)\xi\right\} - \left\{\beta\frac{n_{fwC}n_{cpwc}}{n_{fwC}+n_{cpwC}} + \left(\sqrt{\beta\frac{n_{fwC}n_{cpwc}}{n_{fwC}+n_{cpwC}}}\right)\xi\right\} + \left\{SA.\,n_{pwC} + \left(\sqrt{SA.\,n_{pwC}}\right)\xi\right\} \quad (31)$$

$$\frac{\partial n_{fsed}}{\partial t} = \left\{k_{set}\gamma n_{fsed} + \left(\sqrt{k_{set}\gamma n_{fsed}}\right)\xi\right\} - \; k_{resus}n_{fsed} + \left(\left(1 - \frac{N_{sed}}{N_{sedmax}}\right)n_{fsed}.\,\mu_{fsed}\right) - \left\{a.\,n_{fsed} + \left(\sqrt{a.\,n_{fsed}}\right)\xi\right\} - \left\{\beta\frac{n_{fsed}n_{psed}}{n_{fsed}+n_{psed}} + \left(\sqrt{\beta\frac{n_{fsed}n_{psed}}{n_{fsed}+n_{psed}}}\right)\xi\right\} - \left\{\beta\frac{n_{fsed}n_{cpsed}}{n_{fsed}+n_{cpsed}} + \left(\sqrt{\beta\frac{n_{fsed}n_{cpsed}}{n_{fsed}+n_{cpsed}}}\right)\xi\right\} + \left\{SA.\,n_{psed} + \left(\sqrt{SA.\,n_{psed}}\right)\xi\right\} \quad (32)$$

$$\frac{\partial n_{pwC}}{\partial t} = D\frac{\partial^2 n_{pwC}}{\partial x^2} - u\frac{\partial n_{pwC}}{\partial x} - \left\{k_{set}n_{pwC} + \left(\sqrt{k_{set}n_{pwC}}\right)\xi\right\} + \frac{k_{resus}}{\gamma}n_{psed} + \left(\left(1 - \frac{N_{wC}}{N_{wcmax}}\right)n_{pwC}.\,\mu_{pwC}\right) - \left\{a.\,n_{pwC} + \left(\sqrt{a.\,n_{pwC}}\right)\xi\right\} + \left\{\beta\frac{n_{fwC}n_{pwC}}{n_{fwC}+n_{pwC}} + \right. \quad (33)$$

$$\left(\sqrt{\beta\frac{n_{fwC}n_{pwC}}{n_{fwC}+n_{pwC}}}\right)\xi\Bigg\} + \Bigg\{\beta\frac{n_{fwC}n_{cpwc}}{n_{fwC}+n_{cpwC}} + \left(\sqrt{\beta\frac{n_{fwC}n_{cpwc}}{n_{fwC}+n_{cpwC}}}\right)\xi\Bigg\} - \{SA.n_{pwC} + (\sqrt{SA.n_{pwC}})\xi\}$$

$$\frac{\partial n_{psed}}{\partial t} = \{k_{set}\gamma n_{psed} + (\sqrt{k_{set}\gamma n_{psed}})\xi\} - k_{resus}n_{psed} + \left(\left(1-\frac{N_{sed}}{N_{sedmax}}\right)n_{psed}.\mu_{psed}\right) - \{a.n_{psed} + (\sqrt{a.n_{psed}})\xi\} + \Bigg\{\beta\frac{n_{fsed}n_{psed}}{n_{fsed}+n_{psed}} + \left(\sqrt{\beta\frac{n_{fsed}n_{psed}}{n_{fsed}+n_{psed}}}\right)\xi\Bigg\} + \Bigg\{\beta\frac{n_{fsed}n_{cpsed}}{n_{fsed}+n_{cpsed}} + \left(\sqrt{\beta\frac{n_{fsed}n_{cpsed}}{n_{fsed}+n_{cpsed}}}\right)\xi\Bigg\} - \{SA.n_{psed} + (\sqrt{SA.n_{psed}})\xi\} \tag{34}$$

$$\frac{\partial n_{cpwc}}{\partial t} = D\frac{\partial^2 n_{cpwC}}{\partial x^2} - u\frac{\partial n_{cpwC}}{\partial x} - \{k_{set}n_{cpwC} + (\sqrt{k_{set}n_{cpwC}})\xi\} + \frac{k_{resus}}{\gamma}n_{cpsed} + \left(\left(1-\frac{N_{wC}}{N_{wcmax}}\right)n_{cpwC}.\mu_{cpwC}\right) - \{a.n_{cpwC} + (\sqrt{a.n_{cpwC}})\xi\} + \Bigg\{\beta\frac{n_{cpwC}n_{cwC}}{n_{cpwC}+n_{cwC}} + \left(\sqrt{\beta\frac{n_{cpwC}n_{cwC}}{n_{cpwC}+n_{cwC}}}\right)\xi\Bigg\} + \Bigg\{\beta\frac{n_{cwC}n_{pwc}}{n_{cwC}+n_{pwC}} + \left(\sqrt{\beta\frac{n_{cwC}n_{pwc}}{n_{cwC}+n_{pwC}}}\right)\xi\Bigg\} - \{SA.n_{cpwC} + (\sqrt{SA.n_{cpwC}})\xi\} \tag{35}$$

$$\frac{\partial n_{cpsed}}{\partial t} = \{k_{set}\gamma n_{cpsed} + (\sqrt{k_{set}\gamma n_{cpsed}})\xi\} - k_{resus}n_{cpsed} + \left(\left(1-\frac{N_{sed}}{N_{sedmax}}\right)n_{cpsed}.\mu_{cpsed}\right) - \{a.n_{cpsed} + (\sqrt{a.n_{cpsed}})\xi\} + \Bigg\{\beta\frac{n_{cpsed}n_{csed}}{n_{cpsed}+n_{csed}} + \left(\sqrt{\beta\frac{n_{cpsed}n_{csed}}{n_{cpsed}+n_{csed}}}\right)\xi\Bigg\} + \Bigg\{\beta\frac{n_{csed}n_{psed}}{n_{csed}+n_{psed}} + \left(\sqrt{\beta\frac{n_{csed}n_{psed}}{n_{csed}+n_{psed}}}\right)\xi\Bigg\} - \{SA.n_{cpsed} + (\sqrt{SA.n_{cpsed}})\xi\} \tag{36}$$

$$\frac{\partial n_{cwC}}{\partial t} = D\frac{\partial^2 n_{cwC}}{\partial x^2} - u\frac{\partial n_{cwC}}{\partial x} - \{k_{set}n_{cwC} + (\sqrt{k_{set}n_{cwC}})\xi\} + \frac{k_{resus}}{\gamma}n_{csed} + \left(\left(1-\frac{N_{wC}}{N_{wcmax}}\right)n_{cwC}.\mu_{cwC}\right) - \{a.n_{cwC} + (\sqrt{a.n_{cwC}})\xi\} - \Bigg\{\beta\frac{n_{cwC}n_{pwC}}{n_{cwC}+n_{pwC}} + \left(\sqrt{\beta\frac{n_{cwC}n_{pwC}}{n_{cwC}+n_{pwC}}}\right)\xi\Bigg\} - \Bigg\{\beta\frac{n_{cwC}n_{cpwc}}{n_{cwC}+n_{cpwC}} + \left(\sqrt{\beta\frac{n_{cwC}n_{cpwc}}{n_{cwC}+n_{cpwC}}}\right)\xi\Bigg\} + \{SA.n_{cpwC} + (\sqrt{SA.n_{cpwC}})\xi\} \tag{37}$$

$$\frac{\partial n_{csed}}{\partial t} = \{k_{set}\gamma n_{csed} + (\sqrt{k_{set}\gamma n_{csed}})\xi\} - k_{resus}n_{csed} + \left(\left(1-\frac{N_{sed}}{N_{sedmax}}\right)n_{csed}.\mu_{csed}\right) - \{a.n_{csed} + (\sqrt{a.n_{csed}})\xi\} - \Bigg\{\beta\frac{n_{csed}n_{psed}}{n_{csed}+n_{psed}} + \tag{38}$$

$$\left(\sqrt{\beta \frac{n_{csed}n_{psed}}{n_{csed}+n_{psed}}}\right)\xi\right\} - \left\{\beta \frac{n_{csed}n_{cpsed}}{n_{csed}+n_{cpsed}} + \left(\sqrt{\beta \frac{n_{csed}n_{cpsed}}{n_{csed}+n_{cpsed}}}\right)\xi\right\} + \left\{SA.n_{cpsed} + \left(\sqrt{SA.n_{cpsed}}\right)\xi\right\}$$

S.2 The detail description of schematic model and equation from S1 to S38 is given in Gothwal and Thatikonda, 2017

S.2.1 Model development

The model is developed for steady state one-dimensional advection-dispersion dominated transport of fluoroquinolones and its resistant bacteria in the riverine environment. The model comprises of physical processes such as advection, dispersion, adsorption, degradation, settling, resuspension, diffusion, to formulate the transport of antibiotic, metals, suspended solids, dissolved organic matter, particulate organic matter, bacteria and antibiotic resistance. The schematic of the model is presented in Figure S1. The state variables in the model are: concentrations of fluoroquinolone (A), heavy metal (M), total suspended solid (TSS), particulate organic matter (POM), dissolved organic matter (DOM), total bacterial culture (N) in the water column and sediment bed.

S.2.2 Partitioning in water and sediment column

$$A_{fd,wc} = \frac{A_{wC}}{1 + K_{d,DOM,A}DOM_{wC} + K_{d,Solid,A}TSS_{wC}} \quad \text{(S1)}$$

$$A_{d,wc} = \frac{A_{wC}K_{d,DOM,A}DOM_{wC}}{1 + K_{d,DOM,A}DOM_{wC} + K_{d,Solid,A}TSS_{wC}} \quad \text{(S2)}$$

$$A_{p,wc} = \frac{A_{wC}\ K_{d,Solid,A}TSS_{wC}}{1 + K_{d,DOM,A}DOM_{wC} + K_{d,Solid,A}TSS_{wC}} \quad \text{(S3)}$$

$$M_{fd,wc} = \frac{M_{wC}}{1 + K_{d,DOM,M}DOM_{wC} + K_{d,Solid,M}TSS_{wC}} \quad \text{(S4)}$$

$$M_{d,wc} = \frac{M_{wC}K_{d,DOM,M}DOM_{wC}}{1 + K_{d,DOM,M}DOM_{wC} + K_{d,Solid,M}TSS_{wC}} \quad \text{(S5)}$$

$$M_{p,wc} = \frac{M_{wC}\ K_{d,Solid,M}TSS_{wC}}{1 + K_{d,DOM,M}DOM_{wC} + K_{d,Solid,M}TSS_{wC}} \quad \text{(S6)}$$

S.2.3 The partitioning equation in bed sediment is accounted for porosity (ø) and the equations are as follows:

$$A_{fd,sed} = \frac{A_{sed}.\ ø}{ø + K_{d,DOM,A}DOM_{sed} + K_{d,Solid,A}TSS_{sed}} \quad \text{(S7)}$$

$$A_{d,sed} = \frac{A_{sed}K_{d,DOM,A}DOM_{sed}}{ø + K_{d,DOM,A}DOM_{sed} + K_{d,Solid,A}TSS_{sed}} \quad \text{(S8)}$$

$$A_{p,sed} = \frac{A_{sed}\ K_{d,Solid,A}TSS_{sed}}{ø + K_{d,DOM,A}DOM_{sed} + K_{d,Solid,A}TSS_{sed}} \quad \text{(S9)}$$

$$M_{fd,sed} = \frac{M_{sed}.\ ø}{ø + K_{d,DOM,M}DOM_{sed} + K_{d,Solid,M}TSS_{sed}} \quad \text{(S10)}$$

$$M_{d,sed} = \frac{M_{sed}K_{d,DOM,M}DOM_{sed}}{ø + K_{d,DOM,M}DOM_{sed} + K_{d,Solid,M}TSS_{sed}} \quad \text{(S11)}$$

$$M_{p,sed} = \frac{M_{sed}\ K_{d,Solid,M}TSS_{sed}}{ø + K_{d,DOM,M}DOM_{sed} + K_{d,Solid,M}TSS_{sed}} \quad \text{(S12)}$$

S.2.4 Growth of bacteria

$$\mu_{f,wc} = \begin{cases} \mu_{maxwc}\left(\frac{k_i}{k_i + M_{fd,wc}}\right)\left(1 - \frac{A_{fd,wc}}{MIC}\right)\left(\frac{DOM_{wc}}{K_s + DOM_{wc}}\right), & A_{fd} < MIC \\ 0, & A_{fd} \geq \text{MIC} \end{cases} \quad \text{(S13)}$$

$$\mu_{p,wc} = \mu_{maxwc}\left(\frac{DOM_{wc}}{K_s + DOM_{wc}}\right)\left(\frac{k_i}{k_i + M_{fd,wc}}\right)\left(1 - C_p - X\right)\left(\frac{MIC}{MIC + A_{fd,wc}}\right) \quad \text{(S14)}$$

$$\mu_{c,wc} = \mu_{maxwc} \left(\frac{DOM_{wc}}{K_s + DOM_{wc}}\right)\left(\frac{k_i}{k_i + M_{fd,wc}}\right)(1 - C_c) \left(\frac{MIC}{MIC + A_{fd,wc}}\right) \tag{S15}$$

$$\mu_{cp,wc} = \mu_{maxwc} \left(\frac{DOM_{wc}}{K_s + DOM_{wc}}\right)\left(\frac{k_i}{k_i + M_{fd,wc}}\right)(1 - C_c - C_p - X)\left(\frac{MIC}{MIC + A_{fd,wc}}\right) \tag{S16}$$

$$\mu_{f,sed} = \begin{cases} \mu_{maxsed} \left(\frac{k_i}{k_i + M_{fd,sed}}\right)\left(1 - \frac{A_{fd,sed}}{MIC}\right)\left(\frac{DOM_{sed}}{K_s + DOM_{sed}}\right) \quad , & A_{fd} < MIC \\ 0, & A_{fd} \geq \text{MIC} \end{cases} \tag{S17}$$

$$\mu_{p,sed} = \mu_{maxsed} \left(\frac{DOM_{sed}}{K_s + DOM_{sed}}\right)\left(\frac{k_i}{k_i + M_{fd,sed}}\right)(1 - C_p - X) \left(\frac{MIC}{MIC + A_{fd,sed}}\right) \tag{S18}$$

$$\mu_{c,sed} = \mu_{maxsed} \left(\frac{DOM_{sed}}{K_s + DOM_{sed}}\right)\left(\frac{k_i}{k_i + M_{fd,sed}}\right)(1 - C_c) \left(\frac{MIC}{MIC + A_{fd,sed}}\right) \tag{S19}$$

$$\mu_{cp,sed} = \mu_{maxsed} \left(\frac{DOM_{sed}}{K_s + DOM_{sed}}\right)\left(\frac{k_i}{k_i + M_{fd,sed}}\right)(1 - C_c - C_p - X)\left(\frac{MIC}{MIC + A_{fd,sed}}\right) \tag{S20}$$

S.2.5 Mass balance equations

a) Antibiotics

$$\frac{\partial A_{wC}}{\partial t} = D \frac{\partial^2 A_{wC}}{\partial x^2} - u \frac{\partial A_{wC}}{\partial x} - k_{set} A_{pwc} + \frac{k_{resus}}{\gamma} A_{sed} - \frac{k_{diff}}{h} \left(A_{dwc} + A_{fd,wc} - \frac{A_{d,sed}}{ø} - \frac{A_{fd,sed}}{ø}\right) - K_{xawc} A_{wC} \tag{S21}$$

$$\frac{\partial A_{sed}}{\partial t} = k_{set}\gamma A_{pwc} - k_{resus}A_{sed} - \frac{k_{diff}}{d}\left(\frac{A_{d,sed}}{ø} + \frac{A_{fd,sed}}{ø} - A_{d,wc} - A_{fd,wc}\right) - K_{xased}A_{sed} \quad \text{(S22)}$$

b) Metals

$$\frac{\partial M_{wC}}{\partial t} = D\frac{d^2 M_{wC}}{\partial x^2} - u\frac{\partial M_{wC}}{\partial x} - k_{set}M_{pwc} + \frac{k_{resus}}{\gamma}M_{sed} - \frac{k_{diff}}{h}\left(M_{d,wc} + M_{fd,wc} - \frac{M_{d,sed}}{ø} - \frac{M_{fd,sed}}{ø}\right) - \eta\left(\frac{\mu_{f,wc}\,n_{f,wc}}{Y_f} + \frac{\mu_{p,wc}\,n_{p,wc}}{Y_p} + \frac{\mu_{c,wc}\,n_{c,wc}}{Y_c} + \frac{\mu_{cp,wc}n_{cp,wc}}{Y_{cp}}\right) \quad \text{(S23)}$$

$$\frac{\partial M_{sed}}{\partial t} = k_{set}\gamma M_{pwc} - k_{resus}M_{sed} - \frac{k_{diff}}{d}\left(\frac{M_{d,sed}}{ø} + \frac{M_{fd,sed}}{ø} - M_{d,wc} - M_{fd,wc}\right) - \eta\left(\frac{\mu_{f,sed}\,n_{f,sed}}{Y_f} + \frac{\mu_{p,sed}\,n_{p,sed}}{Y_p} + \frac{\mu_{c\,,sed}n_{c,sed}}{Y_c} + \frac{\mu_{cp,sed}\,n_{cp,sed}}{Y_{cp}}\right) \quad \text{(S24)}$$

c) Total suspended solid

$$\frac{\partial TSS_{wC}}{\partial t} = D\frac{\partial^2 TSS_{wC}}{\partial x^2} - u\frac{\partial TSS_{wC}}{\partial x} - k_{Set}\,TSS_{wC} + \frac{k_{resus}}{\gamma}TSS_{sed} - Z\,K_{H,wc}\,TSS_{wC} \quad \text{(S25)}$$

$$\frac{\partial TSS_{sed}}{\partial t} = k_{Set}\gamma\,TSS_{wC} - k_{resus}TSS_{sed} - ZK_{H,sed}\,TSS_{sed} \quad \text{(S26)}$$

d) Particulate organic matter

$$\frac{\partial(POM_{wc})}{\partial t} = D\frac{\partial^2(POM_{wc})}{\partial x^2} - u\frac{\partial(POM_{wc})}{\partial x} - k_{set}POM_{wc} + \frac{k_{resus}}{\gamma}POM_{sed} - K_{H,wc}\,POM_{wc} + (an_{fwC}) + (an_{pwC}) + (an_{cwC}) + (an_{cpwC}) \quad \text{(S27)}$$

$$\frac{\partial(POM_{sed})}{\partial t} = k_{set}\gamma POM_{wc} - k_{resus}POM_{sed} - K_{H,sed}\, POM_{sed} + (an_{fsed}) + (an_{psed}) + (an_{csed}) + (an_{cpsed}) \quad \text{(S28)}$$

e) Dissolved organic matter

$$\frac{\partial(DOM_{wc})}{\partial t} = D\frac{\partial^2(DOM_{wc})}{\partial x^2} - u\frac{\partial(DOM_{wc})}{\partial x} - \frac{k_{diff}}{h}\,(DOM_{wc} - DOM_{sed}) + K_{H,wc}\, POM_{wc} - \left(\frac{\mu_{f,wc}\, n_{f,wc}}{Y_f} + \frac{\mu_{p,wc}\, n_{p,wc}}{Y_p} + \frac{\mu_{c,wc}\, n_{c,wc}}{Y_c} + \frac{\mu_{cp,wc}\, n_{cp,wc}}{Y_{cp}}\right) \quad \text{(S29)}$$

$$\frac{\partial(DOM_{sed})}{\partial t} = \frac{k_{diff}}{d}\,(DOM_{wc} - DOM_{sed}) + K_{H,sed}\, POM_{sed} - \left(\frac{\mu_{f,sed}\, n_{f,sed}}{Y_f} + \frac{\mu_{p,sed}\, n_{p,sed}}{Y_p} + \frac{\mu_{c,sed}\, n_{c,sed}}{Y_c} + \frac{\mu_{cp,sed}\, n_{cp,sed}}{Y_{cp}}\right) \quad \text{(S30)}$$

f) Susceptible bacteria and resistant bacteria

$$\frac{\partial n_{fwC}}{\partial t} = D\frac{\partial^2 n_{fwC}}{\partial x^2} - u\frac{\partial n_{fwC}}{\partial x} - k_{set}n_{fwC} + \frac{k_{resus}}{\gamma}n_{fsed} + (n_{fwC}.\mu_{fwC}) - (a.n_{fwC}.N_{wC}) - (\beta.n_{fwC}(n_{pwC} + n_{cpwc}) + SA.n_{pwC} \quad \text{(S31)}$$

$$\frac{\partial n_{fsed}}{\partial t} = k_{set}\gamma n_{fsed} - k_{resus}n_{fsed} + (n_{fsed}.\mu_{fsed}) - (a.n_{fsed}.N_{sed}) - (\beta.n_{fsed}(n_{psed} + n_{cpsed}) + SA.n_{psed} \quad \text{(S32)}$$

$$\frac{\partial n_{cwC}}{\partial t} = D\frac{\partial^2 n_{cwC}}{\partial x^2} - u\frac{\partial n_{cwC}}{\partial x} - k_{set}n_{cwC} + \frac{k_{resus}}{\gamma}n_{csed} + (n_{cwC}.\mu_{cwC}) - (a.n_{cwC}.N_{wC}) - (\beta.n_{cwC}(n_{pwC} + n_{cpwc}) + SA.n_{cpwC} \quad \text{(S33)}$$

$$\frac{\partial n_{csed}}{\partial t} = k_{set}\gamma n_{csed} - k_{resus}n_{csed} + (n_{csed}.\mu_{csed}) - (a.n_{csed}.N_{sed}) - \left(\beta.n_{csed}(n_{psed} + n_{cpsed}\right) + SA.n_{cpsed}$$

$$\frac{\partial n_{pwC}}{\partial t} = D\frac{\partial^2 n_{pwC}}{\partial x^2} - u\frac{\partial n_{pwC}}{\partial x} - k_{set}n_{pwC} + \frac{k_{resus}}{\gamma}n_{psed} + \left(n_{pwC}.\mu_{pwC}\right) - \left(a.n_{pwC}.N_{wC}\right) + \left(\beta.n_{pwC}.n_{fwC} + \beta.n_{cpwC}.n_{fwC}\right) - SA.n_{pwC} \tag{S34}$$

$$\frac{\partial n_{psed}}{\partial t} = k_{set}\gamma n_{psed} - k_{resus}n_{psed} + \left(n_{psed}.\mu_{psed}\right) - \left(a.n_{psed}.N_{sed}\right) + \left(\beta.n_{psed}.n_{fsed} + \beta.n_{cpsed}.n_{fsed}\right) - SA.n_{psed}$$

$$\frac{\partial n_{cpwc}}{\partial t} = D\frac{\partial^2 n_{cpwC}}{\partial x^2} - u\frac{\partial n_{cpwC}}{\partial x} - k_{set}n_{cpwC} + \frac{k_{resus}}{\gamma}n_{cpsed} + \left(n_{cpwC}.\mu_{cpwC}\right) - \left(a.n_{cpwC}.N_{wC}\right) + \left(\beta.n_{cpwC}.n_{cwC} + \beta.n_{cwC}.n_{pwC}\right) - SA.n_{cpwC} \tag{S35}$$

$$\frac{\partial n_{cpsed}}{dt} = k_{set}\gamma n_{cpsed} - k_{resus}n_{cpsed} + \left(n_{cpsed}.\mu_{cpsed}\right) - \left(a.n_{cpsed}.N_{sed}\right) + \left(\beta.n_{cpsed}.n_{csed} + \beta.n_{csed}.n_{psed}\right) - SA.n_{cpsed} \tag{S36}$$

Notations:

The following notations are used in this chapter:

A_{wc}= Concentration of antibiotic in water column

A_{fdwc}= Freely dissolved concentration of antibiotic in water column

A_{dwc}= DOM-bound concentration of antibiotic in water column

A_{pwc}= Particulate-bound concentration of antibiotic in water column

A_{sed}= Concentration of antibiotic in sediment bed

A_{fdsed}= Freely dissolved concentration of antibiotic in sediment bed

A_{dsed}= DOM-bound concentration of antibiotic in sediment bed

A_{psed}=Particulate bound concentration of antibiotic in sediment bed

M_{wc}= Concentration of metals in water column

M_{fdwc}= Freely dissolved concentration of metals in water column

M_{dwc}= DOM-bound concentration of metals in water column

M_{pwc}= Particulate-bound concentration of metals in water column

M_{sed}= Concentration of metals in sediment bed

M_{fdsed}= Freely dissolved concentration of metals in sediment

M_{dsed}= DOM-bound concentration of metals in sediment

M_{psed}=Particulate bound concentration of metals in sediment bed

D = Diffusion constant

u = advection velocity

TSS_{sed} = Total suspended solids in sediment bed

TSS_{wc} = Total suspended solids in water column

POM_{sed} = Particulate organic matter in sediment bed

POM_{wc} = Particulate organic matter in water column

DOM_{sed} = Dissolved organic matter in Sediment bed

DOM_{wc} = Dissolved organic matter in water column

Z= Fraction of TSS which can be hydrolyzed

K_{set}= Rettling coefficient for solids;

K_{resus}= Re-suspension coefficient for solids

k_{diff}= Diffusion coefficient or mass transfer coefficient between the water column and pore water bed sediment

K_{xawc}= Antibiotic decay constant due to photolysis in water column

K_{xaset}= Antibiotic decay constant due to photolysis in sediment bed

$K_{d,solids,A}$ = TSS partition coefficient for antibiotic

$K_{d,DOM,A}$ = DOM partition coefficient for antibiotic

$K_{d,DOM,M}$ = DOM partition coefficient for metals

$K_{d,solids,M}$ = TSS partition coefficient for metals

K_{Hwc} = Hydrolysis rate constant for particulate organic matter in water column

K_{Hsed} = Hydrolysis rate constant for particulate organic matter in sediment bed

Ø = porosity of sediment

η = Bacteria substrate utilization efficiency

μ_{maxwc}= Maximum specific growth rate of bacteria in water column

μ_{maxsed}= Maximum specific growth rate of bacteria in sediment bed

μ_{fwc} = Specific growth rate of susceptible bacteria in water column

μ_{fsed} = Specific growth rate of susceptible bacteria in sediment bed

μ_{pwc} = Specific growth rate of plasmid mediated resistant bacteria in water column

μ_{psed} = Specific growth rate of plasmid mediated resistant bacteria in sediment bed

μ_{cwc} = Specific growth rate of chromosome mediated resistant bacteria in water column

μ_{csed} = Specific growth rate of chromosome mediated resistant bacteria in sediment bed

μ_{cpwc}= Specific growth rate of plasmid and chromosome mediated resistant bacteria in water column

μ_{cpsed}= Specific growth rate of plasmid and chromosome mediated resistant bacteria in sediment bed

Y_f = Yield coefficient of susceptible bacteria in water column

Y_p = Yield coefficient of plasmid mediated resistant bacteria

Y_c = Yield coefficient of chromosome mediated resistant bacteria

Y_{cp} = Yield coefficient of plasmid and chromosome mediated resistant bacteria

N_{wc} = Total density of bacterial cells in water column

N_{sed} = Total density of bacterial cells in sediment bed

n_{fwc} = Population of susceptible bacteria in water column

n_{fsed} = Population of susceptible bacteria in sediment bed

n_{pwc} = Population of plasmid mediated resistant bacteria in water column

n_{psed} = Population of plasmid mediated resistant bacteria in sediment bed

n_{cwc} = Population of chromosome mediated resistant becteria in water column

n_{csed} = Population of chromosome mediated resistant becteria in sediment bed

n_{pcwc} = Population of plasmid and chromosome mediated resistant becteria in water column

n_{pcsed} = Population of plasmid and chromosome mediated resistant becteria in sediment bed

k_i = Metal inhibition rate of constant

K_s = Half rate constant

MIC = Minimum inhibitory concentration of antibiotics on bacteria

C_c = Cost of resistant gene when carried on chromosome

C_p = Cost of resistant gene when carried on plasmid

x = Cost plasmid carriage

a=Extrinsic density-dependent death rate of bacteria

SA=Rate of segregation

β=Rate of horizontal transfer of plasmid

γ = Ratio of water depth (or volume) to the depth of the active bed sediment layer (h/d)

η=mg of metal reduced per gm of substrate utilized by bacteria

h=Depth of water column
